# Combination of Chinese and Western Medicine Optimizes the Intestinal Microbiota of Exacerbated Chronic Obstructive Pulmonary Disease in Rats

**DOI:** 10.1155/2021/9975407

**Published:** 2021-09-08

**Authors:** Xiaojun Li, Ya Li, Jing Mao, Qingqing Bian, Yinshuang Xuan, Tingting Shen, Suyun Li

**Affiliations:** ^1^Henan Key Laboratory of Chinese Medicine for Respiratory Disease, Henan University of Chinese Medicine, Zhengzhou 450000, China; ^2^Institute for Respiratory Diseases, The First Affiliated Hospital, Henan University of Chinese Medicine, Zhengzhou 450000, China; ^3^Co-Construction Collaborative Innovation Center for Chinese Medicine and Respiratory Disease by Henan and Education Ministry of P.R. China, Henan University of Chinese Medicine, Zhengzhou 450000, China; ^4^College of Pharmacy, Henan University of Chinese Medicine, Zhengzhou 450000, China

## Abstract

Chronic obstructive pulmonary disease (COPD) changes the structure of the intestinal microbiota and activates the acute exacerbation of COPD (AECOPD). Previous studies showed that the way to treat COPD and AECOPD via combination of Chinese and Western medicine was successful. However, the effect of the intervention on the structure of the intestinal microbiota has not been studied. In this study, we collected feces from model rats following intervention, integrated with Chinese and Western medicine, and used 16S rRNA gene sequencing to clarify the effect on intestinal microbiota. *Methods*. Twenty-five rats were randomized into the control, COPD, AECOPD, Western medicine (moxifloxacin hydrochloride tablets + salbutamol sulfate tablets, MXF/STL), and integrated Chinese and Western medicine (Tong Sai granules + moxifloxacin hydrochloride tablets + salbutamol sulfate tablets + Bu Fei Yi Shen granules + salbutamol sulfate tablets, TMS/FS) groups. Lipopolysaccharide-combined cigarette smoke exposure method was used to simulate the acute exacerbation-stabilization of COPD. Then, the model rats were intervened. *Results*. The intervention of combination Chinese and Western medicine improved the lung function, decreased the C-Reactive Protein (CRP) and Serum Amyloid A (SAA), and relieved pathological damage to the pulmonary alveoli and intestinal mucous of AECOPD rats. The proportion of *Firmicutes*, *TM*7, *Oscillospira*, *Clostridium*, *Ruminococcus*, *Blautia*, *Treponema*, and *Turicibacter* decreased, whereas that of *Bacteroidetes, Proteobacteria, Lactobacillus*, and *Allobaculum* increased via the intervention with the combination of Chinese and Western medicine. *Conclusions*. The intervention with Chinese and Western medicine optimizes the intestinal microbiota structure in AECOPD rat model, which provides a basis for the COPD study in the Chinese medicine.

## 1. Introduction

Chronic obstructive pulmonary disease (COPD) is a common, treatable, and preventable disease, which is characterized by airflow limitation and persistent respiratory symptoms [1]. Acute exacerbations of COPD (AECOPD) negatively impact health, disease progression, and rates of hospitalization/readmission; meanwhile, they are important events in the management of COPD. Recurrent AECOPD is an important factor leading to the deterioration for the patients [2]. A large sample study shows that the prevalence of COPD among Chinese adults aged 40 and over is 13.6% in 2014∼2015 [3]. There are approximately three million deaths annually worldwide [4], which corresponds to around 5.4% of all deaths in 2016 [3]. By 2060, there may be over 5.4 million deaths annually from COPD and related conditions [2], and the prevalence of COPD is expected to rise over the next 40 years [5]. The curative effect of combined Chinese and Western medicine is remarkable for COPD. It shows that the combination treatment has advantages in decreasing the frequency of acute exacerbation, improving lung function, relieving clinical symptoms, improving exercise endurance, and improving quality of life in multicenter, randomized, placebo-controlled trials [6, 7]. However, the structure of the intestinal microbiota during COPD stabilization and acute exacerbation has not been defined.

Smoking, a major risk factor for COPD, can change the microecological structure of the mouth [8], damaging the removal function of mucus in the vagina, and the formation of mucus secretions. When the immune system acts on commensal bacteria, it can cause changes in the composition of the microbiota of COPD patients, resulting in the acute exacerbation of COPD [9]. Smoking can also change the structure of the gut microbiota, causing the intestines to release large numbers of intestinal endotoxins and inflammatory markers. These can be transported to the lower respiratory tract via the circulatory system, and the consequent impaired immune function and inflammation can cause AECOPD [10, 11]. The continuing imbalance between the lung and intestinal microbiota leads to pathological changes [12] in COPD, either directly or indirectly.

Evidence-based research [13] shows that the AECOPD is characterized by superfluous syndromes: phlegm (phlegm-heat, phlegm-dampness) blocking the lung, phlegm and blood stasis mutually obstructing the lung, often with deficiency of qi and yin. The disease is mainly located in the lung during this period. According to the principle of “urgent treatment,” we should remove the heat, remove the phlegm, activate blood circulation, relieve the dyspnea symptoms, and supply qi and yin. Tong Sai granules can be selected for treatment. The stabilization phase is mainly characterized by deficiency syndrome, qi deficiency, qi and yin deficiency, phlegm, and blood stasis. The disease is in the lung and kidney during this period. In accordance with the principle of “slow treatment,” qi should be supplied in the lung and kidney by using the Bu Fei Yi Shen granules. Related research shows that the combination of Chinese and Western medicine has positive effects on the rat model of phlegm-heat syndrome in acute exacerbation and dangerous window stage of COPD [14], which can significantly improve lung function, reduce inflammatory reaction, reduce lung tissue injury, shorten recovery time, and reduce the degree of inflammation in acute exacerbation, and even long-term positive effects. However, the structure of the intestinal microbiota during COPD stabilization and acute exacerbation has not been defined.

The purpose of this study was to evaluate the efficacy of the intervention of combination Chinese and Western medicine in AECOPD rats, clarify the structure of intestinal microbiota in rats during the acute exacerbation and stabilization phase of COPD, and observe the corresponding changes in the structure of intestinal microbiota after drug intervention. We hypothesized that changes in the intestinal microbiota during the acute exacerbation-stabilization phase of COPD are dynamic, and the combined intervention of integrated Chinese and Western medicine plays a role in regulating the intestinal microbiota.

To verify our hypothesis, firstly, we developed a rat model of acute exacerbation-stabilization phase of COPD induced by lipopolysaccharide and cigarette smoke exposure. Secondly, we intervened the rat model by integrating Chinese and Western medicine. Thirdly, we evaluated the efficacy of the intervention of combination Chinese and Western medicine in AECOPD rats. Finally, fresh feces of all rats were collected and sequenced using 16S rRNA. We observed significant differences in beta diversity between the AECOPD group and the COPD group. The proportion of *Firmicutes*, *TM*7, *Oscillospira*, *Clostridium*, *Ruminococcus*, *Blautia*, *Treponema*, and *Turicibacter* decreased, while the *Bacteroidetes, Proteobacteria, Lactobacillus*, and *Allobaculum* increased after the intervention of Chinese and Western medicine. It would provide a basis for the prevention and treatment of COPD with Chinese medicine.

## 2. Materials and Methods

### 2.1. Rats

Twenty-five Sprague-Dawley rats (male, weighing 200 ± 20 g) were purchased from Henan Experimental Animal Center (Henan, China, SYXK (Yu) 2017–0001). The animals were housed in cages with dry bedding and had free access to sterilized food and deionized water under standard conditions of humidity (50 ± 10%), temperature (20 ± 2°C), and light (12 h light/12 h dark cycle). All animals were handled with humane care throughout the experiment.

### 2.2. Medicines and Materials

Tong Sai granules contain *Lepidium apetalum* Willd. 12 g, *Pheretima aspergillum* (E. Perrier) 12 g, *Fritillaria thunbergii* Miq 12 g, *Rheum officinale* Baill. 6 g, *Ephedra sinica* Stapf. 9 g, *Paeonia lactiflora* Pall. 12 g, *Ophiopogon japonicus* (Thunb.) Ker-Gawl. 12 g, and *Ardisia japonica* (Thunb.) Bl. 15 g.

Bu Fei Yi Shen granules contain *Panax ginseng* C. A. Mey. 9 g, *Astragalus membranaceus* (Fisch.) Bge. 15 g, *Lycium barbarum* L. 12 g, *Cornus officinalis* Sieb. et Zucc. 12 g, *Epimedium brevicornum* Maxim 9 g, *Schisandra chinensis* (Turcz.) Baill. 9 g, and *Ardisia japonica* (Thunb.) Bl. 9 g.

Tong Sai granules and Bu Fei Yi Shen granules were prepared by the Department of Pharmacology, affiliated to the First Hospital of Henan University of Chinese Medicine (Zhengzhou, China). They were prepared in fluid extract in accordance with the standard operating procedure and dissolved in 0.9% saline before the intragastric administration. High performance liquid chromatography fingerprint has been performed to identify the main chemical constituents in Bu Fei Yi Shen granules [15].

Moxifloxacin hydrochloride tablets (0.4 g/tablet, BAYER, Sichuan, China) and salbutamol sulfate tablets (2 mg/tablet, Jintan, Jiangsu, China) were powdered and dissolved at a concentration of 1 mg/mL with 0.9% saline before use.

Lipopolysaccharides from *Escherichia coli* O55: B5 (LPS, 100 mg, Sigma, USA) were dissolved at a concentration of 1 mg/mL with 0.9% saline before use.

HongqiQu Filter cigarettes (10 mg tar, 1.0 mg nicotine content, 12 mg CO, Zhengzhou, China) were used.

### 2.3. Reagents

Hematoxylin-eosin (HE) staining: paraffin, Xylene, absolute alcohol, eosin, Neutral balsam, Hematoxylin (Beijing, China).

Enzyme-linked immunosorbent assay (ELISA) kit: Rat CRP (C-Reactive Protein), ELISA Kit (E-EL-R0506c), and Rat SAA (Serum Amyloid A), ELISA Kit (E-EL-R3026) (Elabscience, Wuhan, China).

### 2.4. Instruments

Fine Pointe™ series Pulmonary Function Test system (BUXCO, NY, USA), BS210S Electronic balance (Sartorius, Germany), LDZ5-2 Centrifugal machine (Beijing, China), RM2145 Automatic slicing machine (Leica, Germany), YT-7F8 Water Bath-Slide Drier (Wuhan, China), PM-10AD Optical microscopes and photographic systems (OLYMPUS, JAPAN), Image-Pro Plus 6.0 Professional image analysis software (Media Cybernetics, USA), and Clean bench (Suzhou, China) were used.

### 2.5. COPD Rat Model

Twenty-five male Sprague-Dawley rats were randomly divided into control, COPD, AECOPD, and Western medicine (moxifloxacin hydrochloride tablets + salbutamol sulfate tablets, MXF/STL) and integrated with Chinese and Western medicine (Tong Sai granules + moxifloxacin hydrochloride tablets + salbutamol sulfate tablets + Bu Fei Yi Shen granules + salbutamol sulfate tablets, TMS/FS) groups using the RAND function in EXCEL. With the exception of the control group, the other groups of rats received an LPS drip solution (1 mg/mL, 0.05 mg/100 g body mass) in the nasal cavity, twice per week, as well as cigarette smoke exposure generated from a smoke generator (BUXCO, Wilmington, NC, USA) with concentration of 3000 ± 500 ppm, 30 min per time, twice per day, at interval of ≥3 hour, from week 1 to week 8. Following that, LPS solution (1 mg/mL, 0.2 mg/100 g body mass) was administered to the AECOPD, MXF/STL, and TMS/FS rats by means of a nasal drip, to simulate AECOPD.

### 2.6. Intervention

Starting from day 58, MXF/STL group rats were administered moxifloxacin hydrochloride tablets (0.027 g/kg/d) and salbutamol sulfate tablets (0.41 mg/kg/d) for 6 days and then administrated with salbutamol sulfate tablets for 0.41 mg/kg/d for 7 days; TMS/FS group rats were administrated with Tong Sai granules (7.2 g/kg/d), moxifloxacin hydrochloride tablets (0.027 g/kg/d), and salbutamol sulfate tablets (0.41 mg/kg/d) for 6 days and then administrated with Bu Fei Yi Shen granules (4.84 g/kg/d) and salbutamol sulfate tablets (0.41 mg/kg/d) for 7 days. The control, COPD, and AECOPD group rats were administered with 0.9% saline at 2 mL/d. The dose of drug was calculated using the formula: *D*_rat_ = *D*_human_ × (HI_rat_/HI_human_) × (*W*_human_/*W*_rat_)^2/3^

(*D*: dose, HI: body shape index, HI = *A*/*W*^2/3^, *A*: surface area in m^2^, *W*: body mass) [16]. The drug intervention time of this study was based on reference [14].

### 2.7. Preparation of Specimens

All rats were sacrificed within 24 hours after the last administration, and they were banned from eating for 12 hours but were allowed to drink. On day 71, all rats were anesthetized after intraperitoneal injection of 3% pentobarbital 2.5 mL/kg body weight. When the rats' limb muscles relax and breathing weakens, tracheal intubation was administered, then pulmonary function was tested, and whole blood, lung tissue, intestine, and feces were collected.

#### 2.7.1. Pulmonary Function Test

Forced vital capacity (FVC) and forced expiratory volume 0.3 s (FEV_0.3_) were tested by the Fine Point Pulmonary Function Test system (BUXCO, NY, USA).

#### 2.7.2. Serum

The whole blood was collected by exsanguination of the abdominal aorta abdominal aortic and put in centrifuge at 1500 rpm speed for 15 minutes, and serum was collected and stored in a refrigerator at −80°C. C-Reactive Protein (CRP) and Serum Amyloid A (SAA) levels were detected by enzyme-linked immunosorbent assay (ELISA) (Elabscience, Wuhan, China).

#### 2.7.3. Lung and Intestinal Tissue

After collecting whole blood, the lungs were removed from the thoracic cavity, and about 5 cm of intestines was cut upward from the end of the rectum. The left lungs were washed with saline and filled with 10% neutral buffered formalin via the trachea at the same pressure of 30 cm fixative for 2 hours, and then the left lungs and the intestines were immersed in the 10% neutral buffered formalin for at least 24 hours, respectively.

About 3 mm thickness was cut from the maximum diameter of the left lung lobe and the intestine, respectively, then buried by paraffin, and sliced by 4 *μ*m and stained with hematoxylin-eosin (HE); finally, the pathological changes were observed through an optical microscope, respectively.

#### 2.7.4. Feces Collection and 16S rRNA Gene Amplicon Sequencing

Fresh feces collected from all rats were placed in aseptic frozen tubes, frozen rapidly in liquid nitrogen, and transferred to a refrigerator at −80°C.

PCR amplification of the bacterial 16S rRNA gene V3-V4 region was performed using the forward primer 799 F (5′- ACTCCTACGGGAGGCAGCA -3′) and the reverse primer 1193 R (5′- GGACTACHVGGGTWTCTAAT -3′). Sample-specific 7 bp barcodes were incorporated into the primers for multiplex sequencing. The PCR components contained 5 *μ*L of buffer (5x), 0.25 *μ*L of Fast pfu DNA Polymerase (5 U/*μ*L), 2 *μ*L (2.5 mM) of dNTPs, 1 *μ*L (10 *μ*m) of each Forward and Reverse primer, 1 *μ*L of DNA Template, and 14.75 *μ*L of ddH_2_O. Thermal cycling consisted of initial denaturation at 98°C for 5 min, followed by 25 cycles consisting of denaturation at 98°C for 30 s, annealing at 53°C for 30 s, and extension at 72°C for 45 s, with a final extension of 5 min at 72°C. PCR amplicons were purified with Vazyme VAHTSTM DNA Clean Beads (Vazyme, Nanjing, China) and quantified using the Quant-iT PicoGreen dsDNA Assay Kit (Invitrogen, Carlsbad, CA, USA). After the individual quantification step, amplicons were pooled in equal amounts, and pair-end 2 × 250 bp sequencing was performed using the Illumina MiSeq platform with MiSeq Reagent Kit v3 at Shanghai Personal Biotechnology Co., Ltd (Shanghai, China). Microbiota bioinformatics was performed with QIIME2 2019.4 with slight modification according to the official tutorials (https://docs.qiime2.org/2019.4/tutorials/).

### 2.8. Statistical Analyses

Parts of the data were analyzed using SPSS 22.0 (Chicago, USA), expressed as mean ± standard deviation. One-way ANOVA was used in the intergroup comparison, and the LSD test was used when the variance was uniform; otherwise, Dunnett' s test was used. The significance level was set at *α* = 0.05 (*P* < 0.05). The drawing software used the R 3.5.1 (Zurich, Switzerland), GraphPad Prism 7 (California, USA), and the Adobe Illustrator 22.0 (California, USA).

Sequence data analyses were performed using the QIIME2 and R 3.2.0 (Zurich, Switzerland). Alpha diversity indices were calculated using the amplicon sequence variants (ASV) table in QIIME2. Beta diversity analysis was performed to investigate the structural variation of microbiota across samples using Jaccard metrics, Bray-Curtis metrics, and UniFrac distance metrics and visualized via principal coordinate analysis (PCoA), nonmetric multidimensional scaling (NMDS), and unweighted pair-group method with arithmetic means (UPGMA) hierarchical clustering. The significance of differentiation of microbiota structure among groups was assessed by performing a permutation multivariate analysis of variance (PERMANOVA) using QIIME2. Taxa abundances at the ASV levels were statistically compared among samples or groups by MetagenomeSeq.

## 3. Results

### 3.1. Evaluation of the Efficacy

#### 3.1.1. Pulmonary Function

**Figure 1:**
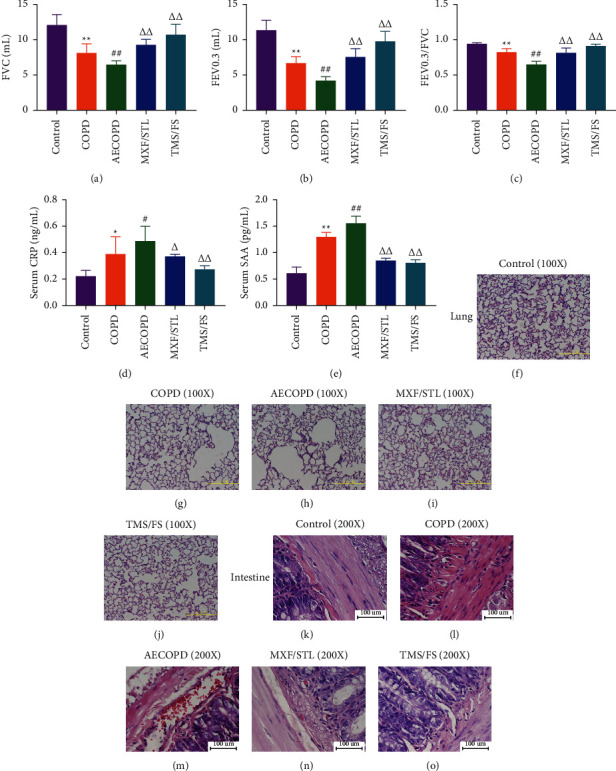
Evaluation of the efficacy of the intervention of combination Chinese and western medicine in AECOPD rats. (a) FVC. (b) FEV_0.3_. (c) FEV_0.3_/FVC. (d) CRP in serum. (e) SAA in serum. The data are expressed as means ± SD, *n* = 5, one-way ANOVA. ^*∗*^*P* < 0.05, compared to control group; ^#^*P* < 0.05, compared to COPD group; ^ΔΔ^*P* < 0.01, compared to AECOPD group. (f)∼(j) Pulmonary tissue pathology stained with HE (100x). (f) Control group, the pulmonary alveoli are complete. (g) COPD group, the structure of the alveoli is incomplete, and some of the alveoli walls are fractured and fused. (h) AECOPD group, the alveoli are badly damaged, and there are large numbers of bubble wall broken and confluent. (i) MXF/STL group, the alveoli structure has been repaired. (j) TMS/FS group, the alveoli structure was repaired to complete. (k)∼(o) Intestinal tissue pathology stained with HE (200x). (k) Control group, there are few inflammatory cells between the intestinal mucous membrane layer and the muscle layer. (l) COPD group, inflammatory cells can be found. (m) AECOPD group, there are large numbers of inflammatory cells and red blood cell immersion. (n) MXF/STL group, some inflammatory cells and red blood can be seen. (o) TMS/FS group, inflammatory cells and red blood cells are almost invisible.

FVC in the COPD group was significantly lower (8.11 mL, *P* < 0.01) than that of the control group (12.04 mL), but significantly higher than that of the AECOPD group (6.41 mL, *P* < 0.01), whereas it increased significantly in the MXF/STL group (9.21 mL, *P* < 0.01) and the TMS/FS group (10.67 mL, *P* < 0.01), compared to the AECOPD group (Figure 1(a)).

FEV_0.3_ in the COPD group was significantly lower (6.63 mL, *P* < 0.01) than that of the control group (11.29 mL), but significantly higher than that of the AECOPD group (4.15 mL, *P* < 0.01). However, it raised significantly in the MXF/STL group (7.50 mL, *P* < 0.01) and the TMS/FS group (9.70 mL, *P* < 0.01), compared to the AECOPD group (Figure 1(b)). The trend of FEV_0.3_/FVC is consistent with the FEV_0.3_ (Figure 1(c), S1).

#### 3.1.2. CRP and SAA Level in Serum

CRP level in the COPD group was significantly higher (0.39 ng/mL, *P* < 0.05) than that of the control group (0.22 ng/mL), but significantly lower than that of the AECOPD group (0.48 ng/mL, *P* < 0.05), while the CRP level decreased significantly in the MXF/STL group (0.37 ng/mL, *P* < 0.05) and the TMS/FS group (0.27 ng/mL, *P* < 0.01), compared to the AECOPD group (Figure 1(d), S2).

SAA in the COPD group is significantly higher (1.29 pg/mL, *P* < 0.01) than that of the control group (0.61 pg/mL), but significantly lower than that of the AECOPD group (1.54 pg/mL, *P* < 0.01). However, the SAA level fell significantly in the MXF/STL group (0.84 pg/mL, *P* < 0.05) and the TMS/FS group (0.80 pg/mL, *P* < 0.05), compared to the AECOPD group (Figure 1(e), S2).

#### 3.1.3. Pathological Changes in Lungs and Intestines

HE staining results showed that the pulmonary alveoli in the control group were complete, while the structure of the alveoli in COPD group was incomplete; some of the alveoli walls were fractured and fused. Furthermore, the alveoli in AECOPD group were badly damaged, and there were large numbers of bubble wall broken and confluent. It was, however, obvious that drug intervention repaired the structure of the alveoli, and the degree of improvement of the alveoli in TMS/FS group was better than that of MXF/STL group (Figures 1(f)–1(j)).

There were few inflammatory cells between the intestinal mucous membrane layer and the muscle layer in the control group, where inflammatory cells could be found in the COPD group; meanwhile, large numbers of inflammatory cells and red blood cells could be seen in the AECOPD group. Some inflammatory cells and red blood cells immersion could be seen in the MXF/STL group, but they were invisible in the TMS/FS group (Figures 1(k)–1(o)).

### 3.2. Information Analysis of the Intestinal Microbiota

#### 3.2.1. Sequence Quality, Length, and Species Taxon Count

**Figure 2:**
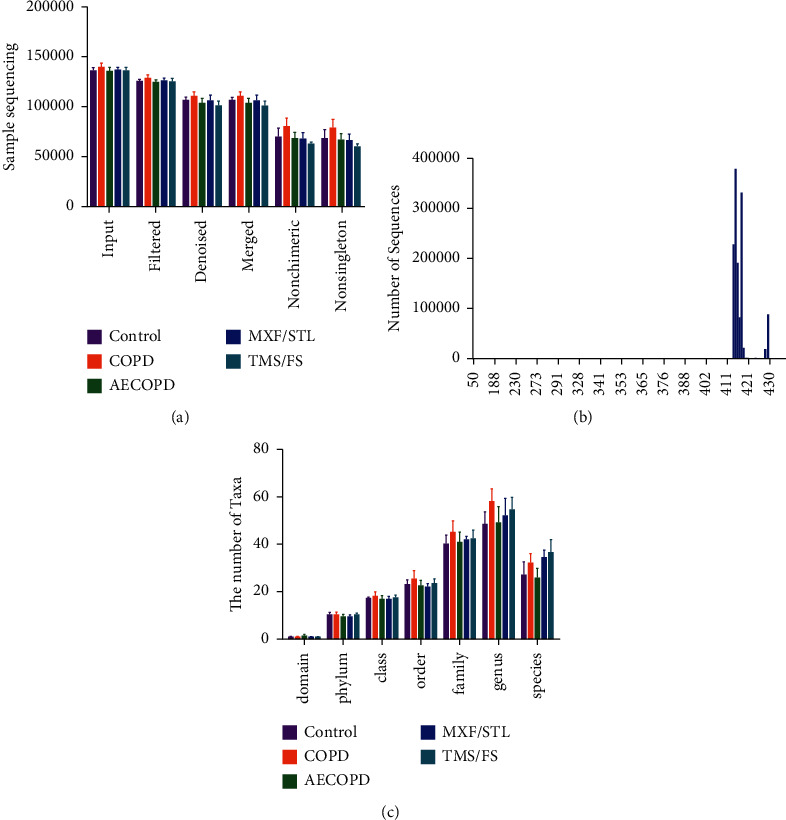
(a) Sequencing quantity of samples in each group. (b) The sequence length distribution. The abscissa indicates the length of the sequence, and the ordinate indicates the number of sequences. (c) The number of taxa. The abscissa indicates classification levels, and the ordinate indicates the number of ASV in each group. Different groups were marked by different colors, and the height of the column corresponds to the number of ASV.

Raw sequence data were demultiplexed, quality filtered, denoised, merged, and chimera removed. A total of 1693888 nonsingleton ASV remained after the preprocessing (Figure 2(a), S3). The distribution range of sequencing length was between 50 bp and 433 bp, and the average length was 411 bp (Figure 2(b), S4). From the six classification levels, there were a total of 4398 taxon (Figure 2(c), S5).

#### 3.2.2. Analysis of the Alpha Diversity Indices

**Figure 3:**
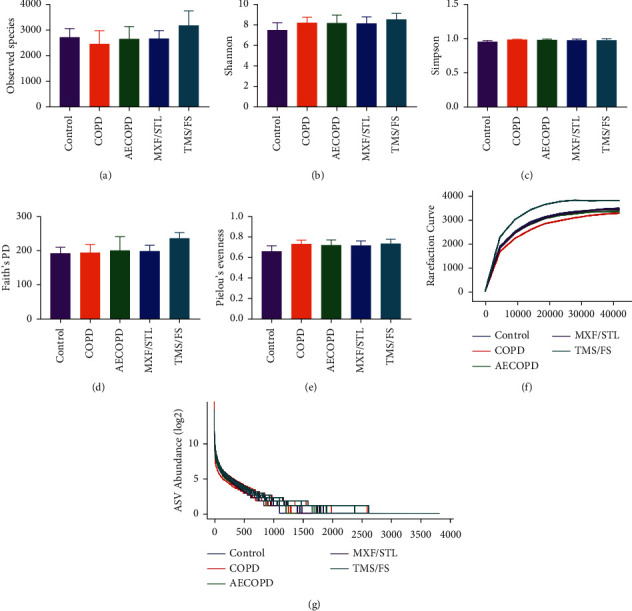
Alpha diversity index distribution in each group. (a) Observed species richness. (b) Shannon index. (c) Simpson index. (d) Faith's phylogenetic diversity index. (e) Pielou's evenness index. The abscissa indicates each group, and the ordinate indicates the index value. The data expressed by means ± SD, *n* = 5, one-way ANOVA. (f) Rarefaction curve. The abscissa indicates the draw depth, and the ordinate indicates the median of the chao1 index calculated 10 times, and the curve is flat, indicating that the sequencing results are sufficient to reflect the diversity contained in the current sample. (g) Rank abundance curve. The abscissa sorts the abundance size, the ordinate indicates the values of each ASV in this group converted by Log2, and the flatter the line, the smaller the abundance difference between the ASVs in the community.

The observed species richness was 2277 in the control group, 2675 in the COPD group, 2638 in the AECOPD group, 2652 in MXF/STL group, and 3162 in TMS/FS group. There was no significant difference among the groups (*P* > 0.05, Figure 3(a)). The Shannon index was 7.887 in the control group, 7.655 in the COPD group, 8.157 in the AECOPD group, 8.118 in MXF/STL group, and 8.510 in TMS/FS group. There was no significant difference among the groups (*P* > 0.05, Figure 3(b)). The Simpson index was 0.980 in the control group, 0.938 in the COPD group, 0.979 in the AECOPD group, 0.975 in MXF/STL group, and 0.977 in TMF/FS group. There was no significant difference among the groups (*P* > 0.05, Figure 3(c)). Faith's Phylogenetic Diversity index was 177 in the control group, 200 in the COPD group, 199.6 in the AECOPD group, 197.6 in MXF/STL group, and 235 in TMS/FS group. There was no significant difference among the groups (*P* > 0.05, Figure 3(d)). Pielou's Evenness index was 0.708 in the control group, 0.673 in the COPD group, 0.718 in the AECOPD group, 0.714 in MXF/STL group, and 0.733 in TMS/FS group. There was no significant difference among the groups (*P* > 0.05, Figure 3(e), S6).

Rarefaction curve describes the trend of the sample Chao1 index with the draw depth. The abscissa indicates the draw depth, and the ordinate indicates the median of the Chao1 index calculated 10 times. We can see the curves that when the abscissa (sequencing depth) is about 4000, and the ordinate (Chao1 index, after 10 times' calculation) is about 4000, they tend to be flat (Figure 3(f)).

Rank abundance curve visually reflects the number of high abundance and rare ASVs in the microbiota. The abscissa indicates the order of ASVs arranged by abundance size, and the ordinates indicated the values of each ASV in this group converted by Log2. We can observe that the TMS/FS groups were longer and flatter above the horizontal coordinates (Figure 3(g)).

#### 3.2.3. Analysis of the Beta Diversity Indices

**Figure 4:**
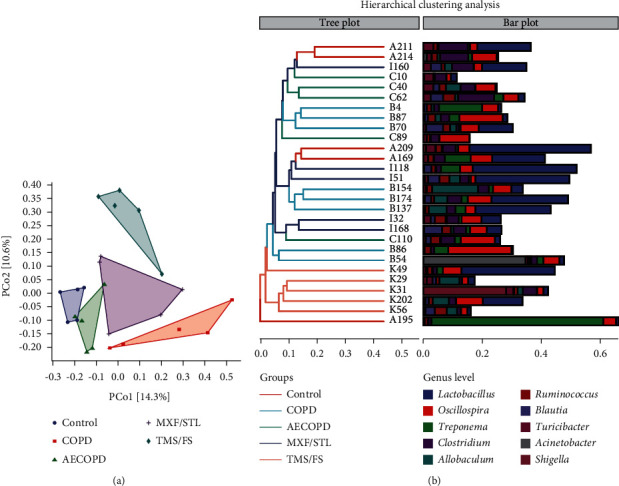
Analysis of the beta diversity indices of ASV in each group. (a) PCoA analysis based on Bray-Curtis distance matrix algorithm. Each dot represents a sample, and dots of different colors indicate different groups. The percentage in parentheses of the axis represents the proportion of the sample distance matrix that can be explained by the corresponding axis. The closer the distance, the more similar the ASV composition of the two samples in the corresponding dimensions. (b) Hierarchical clustering based on the unweighted pair-group method with arithmetic means (UPGMA). The panel on the left is a hierarchical clustering tree map, where the samples were clustered according to the similarity between each other, and the shorter the branch length between the samples, the greater the similarity; the panel on the right is the histogram of the genus level microbiota.

According to the abundance of information in ASVs in the sample and the evolutionary relationship between representative sequences, we used the Bray-Curtis distance algorithm to form the sample difference distance matrix. As shown in Figure 4(a)S7), the matrix space of the two dimensions explained the differences by 14.3% and 10.6%, respectively. There was a significant difference in the ASV distance matrix between the COPD group and the control group (*P* < 0.01). There was a significant difference in the ASV distance matrix between the AECOPD group and the COPD group (*P* < 0.05). The difference in the ASV distance matrix during the MXF/STL, TMS/FS, and AECOPD groups was significant (*P* < 0.05), respectively (S8).

Principal coordinate analysis (PCoA) is one of the most classical multidimensional scaling sorting methods. It can reflect the sample difference distance in the distance matrix to the maximum extent. As shown in Figure 4(b), the ASVs of the TMS/FS and COPD groups clustered better, and the ASVs of MXF/STL and AECOPD groups clustered better at the genus level.

#### 3.2.4. Taxonomic Composition Analysis

The top 10 microbiota with a proportion of more than 1% include *Firmicutes, Bacteroidetes, Proteobacteria*, and *TM*7 at the phylum level (S9).

**Figure 5:**
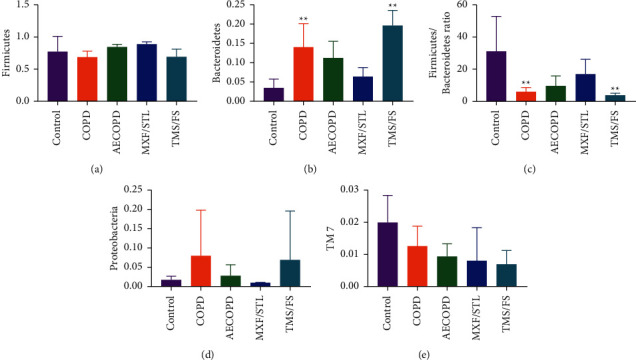
The top 10 microbiota with a proportion of more than 1% in each group at the phylum level. The abscissa represents each group, and the ordinate represents the proportion. The data were expressed as means ± SD, *n* = 5, and one-way ANOVA. ^*∗*^*P* < 0.05, compared to the control group; ^#^*P* < 0.05, compared to the COPD group.

The proportion of *Firmicutes* in the control group was 76.89%, which declined in the COPD group (68.31%) and climbed in the AECOPD group (84.08%). It is up to 88.52% in the MXF/STL group, but down to 68.83% in the TMS/FS group (Figure 5(a)).

The proportion of *Bacteroidetes* in the control group was 3.39%, which climbed in the COPD group (13.96%, *P* < 0.01) and AECOPD group (11.16%), whilst dropping to 6.37% in the MXF/STL group; however, it returned to 19.58% in the TMS/FS group (Figure 5(b)).

The *Firmicutes/Bacteroidetes* (F/B) ratio was 30.87 in the control group, which dropped significantly in the COPD group (5.87, *P* < 0.01), but it went up to 9.34 in the AECOPD group. After the intervention, the F/B ratio becomes 16.65 in the MXF/STL group and 3.68 in the TMS/FS group (Figure 5(c)).

*Proteobacteria* level was 1.67% in the control group, which climbed in the COPD group (7.93%) and declined in the AECOPD group (2.69%). It dropped to 0.90% in the MXF/STL group, but it reached 6.82% in the TMS/FS group (Figure 5(d)).

*TM*7 took a 1.99% share in the control group, which was 1.24% in the COPD group and 0.92% in the AECOPD group. The proportion dropped to 0.80% in the MXF/STL group, and it went down slightly to 0.69% in the TMS/FS group (Figure 5(e)).

The top 10 microbiota with a proportion of more than 1% include *Lactobacillus, Oscillospira, Clostridium, Allobaculum, Ruminococcus, Blautia, Treponema*, and *Turicibacter* at the genus level (S10).

**Figure 6:**
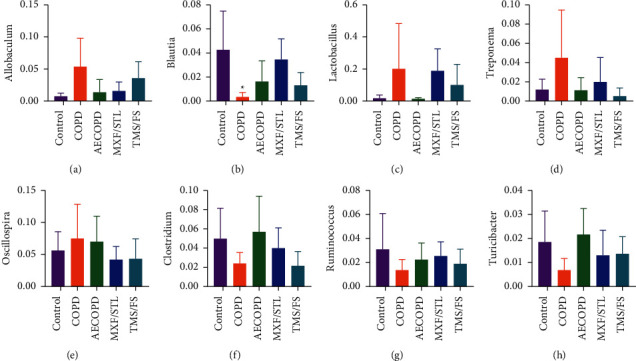
The top 10 microbiota with a proportion of more than 1% in each group at the genus level. The abscissa represents each group, and the ordinate represents the proportion. The data were expressed as means ± SD, *n* = 5, and one-way ANOVA. ^*∗*^*P* < 0.05, compared to the control group; ^#^*P* < 0.05, compared to the COPD group.

The proportion of *Allobaculum* was 0.67% in the control group, which surged in the COPD group (5.29%) but declined in the AECOPD group (1.33%). The proportion increased in the MXF/STL group (1.54%) and went up to 3.52% in the TMS/FS group (Figure 6(a)).

The proportion of *Blautia* was 4.25% in the control group, which decreased significantly in the COPD group (0.31%, *P* < 0.05, Figure 6(b)) but increased in the AECOPD group (1.61%). The proportion showed an upregulation in the MXF/STL group (3.45%) and a downregulation in the TMS/FS group (1.28%).

The proportions of *Lactobacillus* and *Treponema* were low in the control group (1.65%, 1.16%, respectively), and they surged in the COPD group (19.88%, 4.48%, respectively) but declined in the AECOPD group (1.25%, 1.1%, respectively). The proportions showed upregulation in the MXF/STL group (18.64%, 1.96%, respectively) and downregulation in the TMS/FS group (9.94%, 0.49%, respectively, Figures 6(c) and 6(d)).

The proportion of *Oscillospira* in the COPD group was 7.46%; it was higher than that in the control and AECOPD groups (5.58%, 6.97%, respectively). The proportion dropped in the MXF/STL group (4.16%) and the TMS/FS group (4.29%) (Figure 6(e)).

The proportion of *Clostridium, Ruminococcus*, and *Turicibacter* in the COPD group was 2.37%, 1.34%, and 0.67%, respectively, which were lower than that of the control group (4.97%, 3.07%, and 1.84%, respectively) and AECOPD group (5.66%, 2.22%, and 2.16%, respectively). The proportion of *Clostridium* and *Turicibacter* was decreased (3.97%, 1.29%, respectively), whereas *Ruminococcus* was increased (2.51%) in the MXF/STL group. The proportions of *Clostridium* and *Ruminococcus* showed downregulation (2.12%, 1.87%, respectively), but *Turicibacter* showed upregulation (1.35%) in the TMS/FS group (Figures 6(f)–6(h)).

## 4. Discussion

COPD is characterized by airflow limitation and persistent respiratory symptoms [1]. According to the Chinese medicine theory, COPD belongs to the category of cough or lung distension. Documented clinical studies have verified that integrated Chinese and Western medicine has good effects on COPD, such as decreasing the frequency of acute exacerbation, improving lung function, relieving clinical symptoms, and improving exercise endurance [6, 7, 17].This study shows that the intervention of integrated Chinese and Western medicine in AECOPD rats can not only reduce the pathological injury of alveoli, but also reduce the inflammatory infiltration of intestinal mucosa by increasing FVC, FEV_0.3_, and FEV_0.3_/FVC, as well as reducing CRP and SAA in serum.

Besides, the intestinal microbiota structure of COPD rats will change in the acute exacerbation-stable period, which can be regulated via the intervention of integrated Chinese and Western medicine.

COPD changes the structure of the intestinal microbiota in rats. Compared to the control group, *Bacteroidetes, Proteobacteria, Lactobacillus, Oscillospira, Allobaculum*, and *Treponema* increased in the COPD group, which suggests that *Bacteroidetes, Proteobacteria, Lactobacillus, Oscillospira, Allobaculum*, and *Treponema* may be involved in the pathogenesis of COPD as copathogenic factors.

Different pathological phases of COPD lead to the structure change of the intestinal microbiota in rats. Compared to COPD stabilized conditions, the proportion of *Firmicutes, Clostridium, Ruminococcus, Blautia*, and *Turicibacter* increased under exacerbation conditions, suggesting that *Firmicutes, Clostridium, Ruminococcus, Blautia*, and *Turicibacter* may be risk factors for AECOPD.

The structure of the intestinal microbiota in AECOPD rats can be regulated via intervention with a combination of Chinese and Western medicine. Compared to that in the AECOPD group, the proportion of *Firmicutes, Lactobacillus, Allobaculum, Ruminococcus, Blautia*, and *Treponema* increased after the intervention of Western medicine, whereas the proportion of *Bacteroidetes, Proteobacteria, TM*7*, Oscillospira, Clostridium*, and *Turicibacter* decreased after the intervention of Western medicine. The proportion of *Firmicutes, TM*7*, Oscillospira, Clostridium, Ruminococcus, Blautia, Treponema*, and *Turicibacter* decreased after the intervention with a combination of Chinese and Western medicine, whereas that of *Bacteroidetes, Proteobacteria, Lactobacillus*, and *Allobaculum* increased after the intervention with a combination of Chinese and Western medicine.

Antibiotic-induced depletion of intestinal microbiota diversity has been widely implicated as a risk factor for infectious diseases [18]. Evidence supports the fact that the drug was the biggest factor affecting changes in fecal microbiota [19]. Our data suggest that the intervention with a combination of Chinese and Western medicine promotes the optimization of the structure of the intestinal microbiota in rats.

Smoking is an independent risk factor for the acute exacerbation of COPD, which is characterized by an increased abundance of *Bacteroides* in a study of healthy smokers [20] and decreased F/B ratio compared with the fecal microbiota in nonsmokers [21]. Chronic inflammation of the airway leads to increased mucus secretions, thus causing narrowing of the airway and restricted breathing. This leads to an increase in the oxygen consumption of the body, which eventually leads to the body being in a low oxygen state. The F/B ratio of the intestinal microbiota in rats in the low oxygen level exposure group was lower than that of the control group [22]. Our data are consistent with documented studies. A small human study analyzed the association between intestinal bacterial composition and cardiopulmonary health indicators in 38 healthy young subjects and showed that the F/B ratio was associated with the maximum oxygen consumption of the subjects [23]. Additionally, evidence has shown that Tibetans have lower F/B ratios due to their long-term exposure to low oxygen levels [24]. Our data shows that the Western medicine increases the F/B ratio, which suggests that the Western medicine therapy regulates the intestinal microbiota and relieves the hypoxic state of exacerbation in COPD rats.

The abundance of *Proteobacteria* is the main factor affecting the functional variation of the human intestinal microbiota [25], and its expansion can be used as a feature of intestinal epithelial dysfunction [26]. Random blood glucose was negatively correlated with *Lactobacillus* and *Turicibacter* but positively correlated with *Ruminococcus* and *Allobaculum* by Simpson's diversity index [27]. *Clostridium* is a member of the normal intestinal microbiota in humans, and the irregular use of antibiotics can lead to intestinal microbiota disorders. *Oscillospira* is associated with a low body mass index in humans and in inflammatory patients, which contributes to the formation of secondary bile acids that are known to protect against infection with *Clostridium* difficile [28]. *E. coli* Nissle 1917 can produce more C18-3OH, and the increase in the abundance of *Allobaculum*. This, in turn, is related to the increased concentration of C18-3OH in the colon, which can exert an anti-inflammatory mechanism by activating peroxisome proliferator activated receptor [29]. *Ruminococcaceae* can often be clustered into “enterotypes,” which explains some of the taxonomic variation [19]. A study showed a significant decrease in *Blautia* in obese children, and specific operational taxonomic units in the *Blautia* are associated with inflammatory indicators. Indeed, in vitro experiments confirmed that obesity-related *Blautia* species exhibit anti-inflammatory properties [30]. *Turicibacter* is consistently associated with host genetics. Changes in the bile acid pool driven by Slc10a2 genetic variation and concomitant changes in expression affect the community structure of gut microbiota and influence the ability of *Turicibacter* to colonize and persist in the intestines [31].

Our research shows that the intervention with a combination of Chinese and Western medicine optimizes the structure of the intestinal microbiota of AECOPD in rats. Our analysis focuses on the diversity of microbiota composition and has exploratory findings because of the small sample size and variances between rats. Further microbiota ecology research should examine functional potential at the metabolomic level. We will investigate using a larger sample size and conduct multiple perspective experiments in future programs.

## 5. Conclusions

The combination of Chinese and Western medicine intervention AECOPD rats can effectively improve pulmonary function and alleviate the pathological damage to their lungs, intestines, as well as the systemic inflammatory response. Furthermore, the diversity of the intestinal microbiota in rats changes dynamically during the acute exacerbation-stabilization phase in COPD, which can be optimized via the intervention of integrated Chinese and Western medicine.

## Data Availability

The data used to support the findings of the study are available in the Supplementary Information files.

## Abbreviations

AECOPD: Acute exacerbations of COPD
ASVs: Amplicon sequence variants
MXF: Moxifloxacin hydrochloride
STL: Salbutamol sulfate
TMS/FS: Tong Sai granules + moxifloxacin hydrochloride tablets + salbutamol sulfate tablets + Bu Fei Yi Shen granules + salbutamol sulfate.

## Supplementary Materials

Supplementary Materials S1: pulmonary function. Evaluation of the FVC, FEV_0.3_, FEV_0.3_/FVC of the intervention of combination Chinese and Western medicine in AECOPD rats. S2: evaluation of the CRP and SAA level in serum of the intervention of combination Chinese and Western medicine in AECOPD rats. S3: sequence quality. Sequencing quantity of samples after the raw sequence data were demultiplexed, quality filtered, denoised, merged, and chimera removed in each group. S4: sequencing length. The distribution range of sequencing length of samples. S5: the number of taxa. The number of taxa at the six classification levels (domain, phylum, class, order, family, genus, species). S6: alpha index. The Observed species, Shannon index, Simpson index, Faith's Phylogenetic Diversity index, and Pielou's Evenness index in each group. S7: PCoA analysis. Principal coordinates analysis (PCoA) based on Bray-Curtis distance matrix algorithm. The principal coordinate analysis method is used to project the distance matrix of the samples, and get the scores of the samples on the first, second and third principal coordinates. S8: difference between groups. PERMANOVA, ANOSIM, and PERMDISP tests were used to verify the differences of PCoA. S9: phylum level proportion. The top 10 microbiota with a proportion of more than 1% include *Firmicutes, Bacteroidetes, Proteobacteria*, as well as *TM*7 at the phylum level. S10: genus level proportion. The top 10 microbiota with a proportion of more than 1% include *Lactobacillus, Oscillospira, Clostridium, Allobaculum, Ruminococcus, Blautia, Treponema*, as well as *Turicibacter* at the genus level.
